# Temporary airway stenting for giant anterior mediastinal tumor biopsy: Two case reports

**DOI:** 10.1016/j.ijscr.2019.10.005

**Published:** 2019-10-07

**Authors:** Ryoichi Matsumoto, Masahiro Mitsuoka, Toshihiro Hashiguchi, Shintaro Yokoyama, Daigo Murakami, Koichi Yoshiyama, Tatsuya Nishi, Masaki Kashihara, Hirofumi Ono, Shinzo Takamori, Yoshito Akagi

**Affiliations:** a Department of Surgery, Yame General Hospital, 540-2, Takatsuka, Yame-shi, Fukuoka 830-0034, Japan; b Department of Surgery, Kurume University, 67, Asahi-machi, Kurume-shi, Fukuoka 830-0011, Japan; c Department of Surgery, Oita Prefecture Saiseikai Hita Hospital, 643-7, Ozimiwa, Hita-shi, Oita 877-1292, Japan

**Keywords:** Anterior mediastinal tumour, Temporary stenting, Central airway obstruction, Case report

## Abstract

**Introduction:**

When the management of an anterior mediastinal tumor requires general anesthesia, airway narrowing and obstruction may occur secondary to muscle relaxation.

**Presentation of Cases:**

Two men (ages, 15 and 36 years) presented with a giant anterior mediastinal tumor and central airway obstruction. We used Dumon stents to effectively secure the airway in both patients. After chemotherapy, stent removal was safely performed in each case because the tumor was substantially smaller.

**Discussion:**

Dumon stents effectively secured the airway. These stents were easily removed after chemotherapy without severe complications.

**Conclusion:**

Temporary stenting is useful in patients with a giant anterior mediastinal tumor who require general anesthesia.

## Highlights

• Anterior mediastinal tumors often cause airway compression.
• General anesthesia for a tumor biopsy can cause fatal airway obstruction.
• Temporary stenting can prevent fatal airway obstruction in anesthetized patients.

## 1 Introduction

The anterior mediastinum is the preferred site of various type of tumors. When the diagnosis or treatment of a huge anterior mediastinal tumor requires general anesthesia, fatal respiratory failure may occur due to airway narrowing and muscle relaxation. We report two cases in which we safely secured the airway using an airway stent while performing a giant anterior mediastinal tumor biopsy under general anesthesia. These cases have been reported in line with the SCARE criteria [1].

## 2 Presentation of cases

**Fig. 1:**
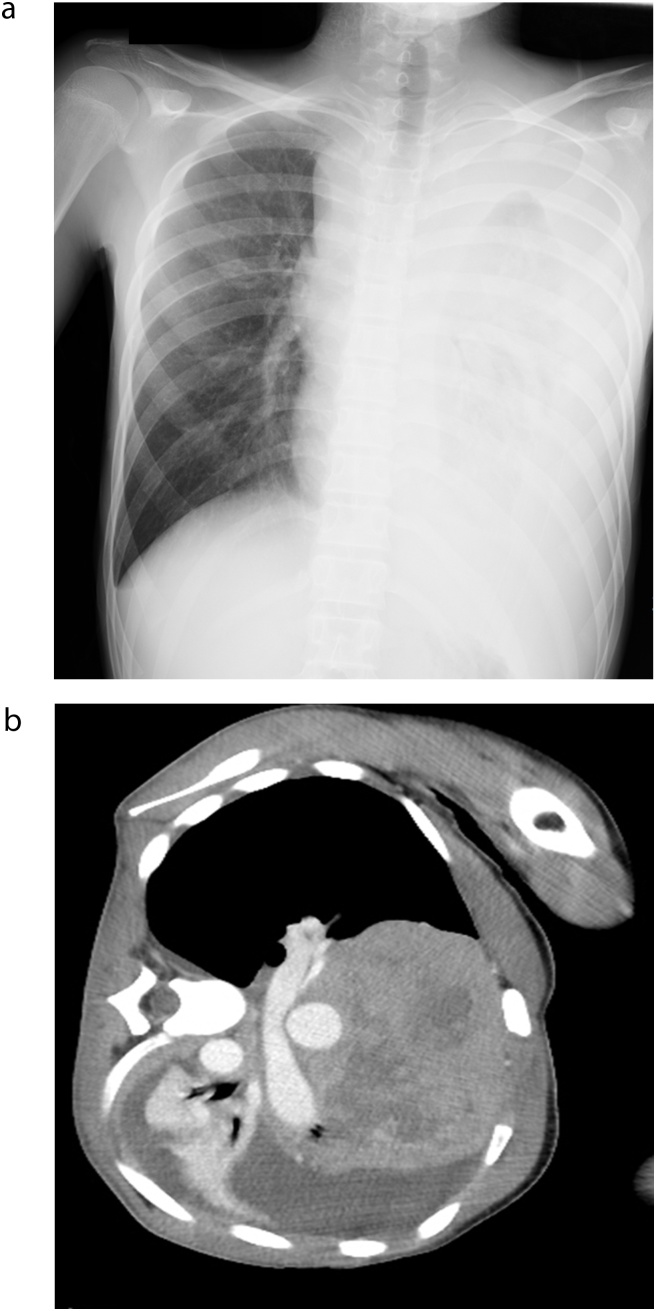
a. Chest X-ray showing an enlarged mediastinal shadow, left atelectasis, and pleural effusion. b. Chest computed tomography showing a large anterior mediastinal tumor, tracheal and cardiovascular compression, left pleural effusion, and atelectasis. The image is rotated because the patient’s respiratory distress was only relieved by placement in the left lateral decubitus position.

**Fig. 2:**
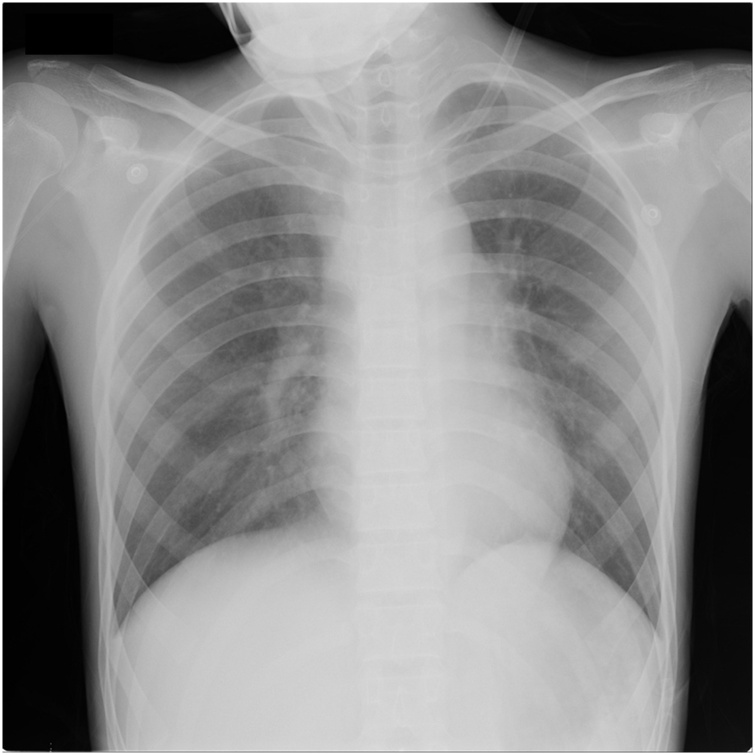
Chest X-ray three months post-chemotherapy showing tumor reduction.

Case 1: A 15-year-old boy presented to our hospital with fever and respiratory distress. On physical examination, his respiratory distress was only relieved by placement in the left lateral decubitus position. The patient’s chest X-rays showed enlarged mediastinal shadows and left lung atelectasis (Fig. 1a). Chest computed tomography (CT) revealed left atelectasis and pleural effusion and a giant anterior mediastinal tumor that markedly compressed the adjacent cardiovascular structures and the trachea (Fig. 1b). We performed a tumor biopsy under general anesthesia and extracorporeal membrane oxygenation (ECMO). Because the patient was 15 years old, there was a possibility that he would require re-positioning during a CT-guided biopsy under local anesthesia. We performed endotracheal intubation and induced general anesthesia after ECMO cannulation. After the biopsy, we could not ventilate the patient, so we started ECMO and intubated his right lateral lung. We placed a straight Dumon stent (Novatech SA, La Ciotat, France) in the left main bronchus and a Dumon Y stent (Novatech SA) at the tracheal bifurcation to maintain his central airways. Postoperatively, the patient’s respiratory condition was stable. We withdrew ECMO support on postoperative day two and mechanical ventilation on postoperative day three. The diagnosis was malignant T-cell lymphoblastic lymphoma, and chemotherapy was administered. Three months later, we could remove the stents because the tumor size was substantially reduced (Fig. 2).

**Fig. 3:**
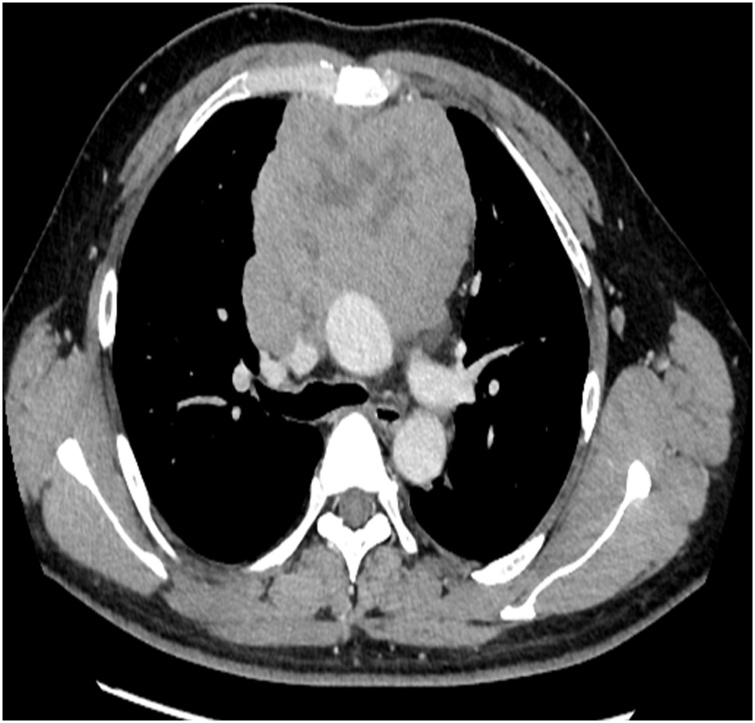
Chest computed tomography showing a large anterior mediastinal tumor and tracheal compression.

**Fig. 4:**
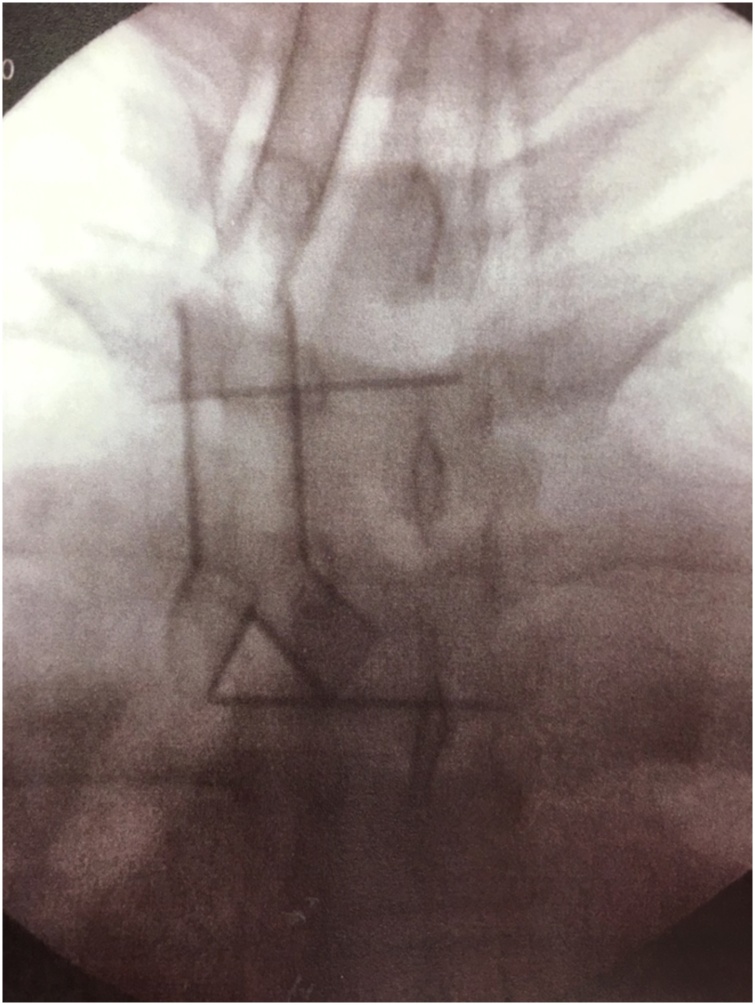
Fluoroscopic image after Dumon Y stent placement at the tracheal bifurcation.

Case 2: A 36-year-old man presented with no particular complaint, and he had no respiratory distress at rest on physical examination, but his abnormal findings were pointed out incidentally. The patient’s chest X-rays showed enlarged mediastinal shadows and no pleural fluid retention. His chest CT showed a giant tumor with a contrast effect in the anterior mediastinum, and, although tracheal and bronchial contractions were observed, complete occlusion had not occurred (Fig. 3). We attempted a tumor biopsy to obtain a definitive diagnosis, and we used local and epidural anesthetics because we felt that general anesthesia could result in increased airway compression. Intraoperative pain control was difficult, and pleural stimulation triggered the patient’s cough reflex. Therefore, surgery was terminated before we could complete the biopsy. Because we still needed a tissue sample for pathological diagnosis, we performed another biopsy with ECMO on standby. Before the biopsy, we placed a Dumon Y stent at the tracheal bifurcation (Fig. 4), performed endotracheal intubation, and confirmed that ventilation was possible. After that, we collected a tumor biopsy sufficient for pathological examination. The tissue diagnosis was dysgerminoma, and chemotherapy was administered. Three months later, we were able to remove the stent because the tumor size was substantially reduced.

## 3 Discussion

Central airway narrowing causes severe dyspnea and may be fatal. However, there are no clear guidelines for the management of a narrow central airway, and only a few reviews on the topic are available [2]. Azizkhan et al. defined severe pediatric tracheobronchial compression as a greater than one-third decrease in luminal area and concluded that affected patients have a substantial risk of total airway obstruction during general anesthesia [3]. Chamberger et al. considered it difficult to predict the extent of airway narrowing secondary to general anesthesia based on CT and lung function data in patients with a mediastinal tumor [4]. Thus, in patients with a giant anterior mediastinal tumor who require general anesthesia, physicians must be prepared to manage cases where it becomes difficult to provide adequate ventilation [2]. Specific measures include auxiliary circulation, appropriate patient positioning, and airway examination to determine whether tracheal tubes can pass the site of stenosis [5].

ECMO is invasive, with the major complication being bleeding secondary to anticoagulation [6]. Consequently, ECMO standby has not been universally accepted. Patient repositioning and tracheal tube adjustment are additional and simple steps, providing room for consideration [7]. However, if the position required for adequate ventilation is different than the position necessary for surgery, the intended procedure cannot be completed.

Therefore, we propose the insertion of a temporary airway stent at the time of induction of general anesthesia that can then be removed when the airway is no longer compressed. Disadvantages of temporary stenting include the possibility of airway injury during stent insertion or removal and stenosis progression due to granulation in cases where the stent is retained for a long period [8]. However, the median time of granulation development is approximately seven months. Thus, if the stent is removed after roughly three months of chemotherapy, as in our cases, late complications may not occur. Compared to other stents, Dumon stents are easy to remove [8]. Further, the probability of airway injury secondary to airway stenting is only 1%. Physicians should not hesitate to place a stent in cases of potentially fatal dyspnea [9,10]. In our cases, if stent placement had been performed before general anesthesia induction, the patients might not have required ECMO or a second surgery.

It should be noted that temporary stenting cannot prevent the compression of large blood vessels. Takeda et al. reported the case of a patient with a large anterior mediastinal tumor who developed severe hypoxemia during general anesthesia secondary to total obstruction of the left main bronchus and right pulmonary artery [11]. In such cases, ventilation is impossible even if the airway is stented, and extracorporeal circulation is required. Further, airway stenting may not prevent cases where ventilation and pulmonary blood flow are compromised at the same time.

## 4 Conclusion

Temporary stenting is useful in patients with a giant anterior mediastinal tumor who require general anesthesia.

## Sources of funding

There is no source of funding for our research.

## Ethical approval

Our case report is exempt from ethnical approval in our institution.

## Consent

Each patient and parents provided consent for the publication of his case, and it is stated in the paper.

## Author contribution

Masahiro Mitsuoka - Manuscript preparation and editing

Toshihiro Hashiguchi - Manuscript preparation

Shintaro Yokoyama - Manuscript preparation

Daigo Murakami - Manuscript preparation

Koichi Yoshiyama - Manuscript preparation

Tatsuya Nishi - Manuscript preparation

Masaki Kashihara - Manuscript preparation

Hirofumi Ono - Manuscript preparation

Shinzo Takamori - Manuscript preparation and editing

Yoshito Akagi- Manuscript preparation and editing

## Registration of research studies

This report is a case report and does not require the application of the Declaration of Helsinki 2013.

## Guarantor

Guarantor: Ryoichi Matsumoto M.D. Masahiro Mitsuoka M.D.

## Provenance and peer review

Not commissioned, externally peer-reviewed

## Declaration of Competing Interest

There is no conflict of interest.
